# Differential Gene Expression Profile Associated with the Abnormality of Bone Marrow Mesenchymal Stem Cells in Aplastic Anemia

**DOI:** 10.1371/journal.pone.0047764

**Published:** 2012-11-05

**Authors:** Jianping Li, Shaoguang Yang, Shihong Lu, Hui Zhao, Jianming Feng, Wenqian Li, Fengxia Ma, Qian Ren, Bin Liu, Lei Zhang, Yizhou Zheng, Zhong Chao Han

**Affiliations:** 1 State Key Laboratory of Experimental Hematology, Institute of Hematology and Hospital of Blood Diseases, Chinese Academy of Medical Sciences and Peking Union Medical College, Tianjin, China; 2 Department of Hematology, Qinghai Provincial People's Hospital, Xining, Qinghai, China; 3 Tianjin Key Laboratory of Food and Biotechnology, School of Biotechnology and Food Science, Tianjin University of Commerce, Tianjin, China; University of Medicine and Dentistry of New Jersey, United States of America

## Abstract

Aplastic anemia (AA) is generally considered as an immune-mediated bone marrow failure syndrome with defective hematopoietic stem cells (HSCs) and marrow microenvironment. Previous studies have demonstrated the defective HSCs and aberrant T cellular-immunity in AA using a microarray approach. However, little is known about the overall specialty of bone marrow mesenchymal stem cells (BM-MSCs). In the present study, we comprehensively compared the biological features and gene expression profile of BM-MSCs between AA patients and healthy volunteers. In comparison with healthy controls, BM-MSCs from AA patients showed aberrant morphology, decreased proliferation and clonogenic potential and increased apoptosis. BM-MSCs from AA patients were susceptible to be induced to differentiate into adipocytes but more difficult to differentiate into osteoblasts. Consistent with abnormal biological features, a large number of genes implicated in cell cycle, cell division, proliferation, chemotaxis and hematopoietic cell lineage showed markedly decreased expression in BM-MSCs from AA patients. Conversely, more related genes with apoptosis, adipogenesis and immune response showed increased expression in BM-MSCs from AA patients. The gene expression profile of BM-MSCs further confirmed the abnormal biological properties and provided significant evidence for the possible mechanism of the destruction of the bone marrow microenvironment in AA.

## Introduction

Aplastic anemia (AA) is generally considered as an immune-mediated bone marrow failure syndrome, characterized by hypoplasia and pancytopenia with fatty bone marrow. Previous investigations have demonstrated that AA manifests as abnormalities of hematopoietic stem cells (HSCs) and the hematopoietic microenvironment, which are mediated by aberrant immune cells and molecules [1], [2]. Aberrant immune cells directly and indirectly destroy stem/progenitor cells in the bone marrow by secreting a variety of immune molecules including interferon-gamma (IFN-γ), tumor necrosis factor-alpha (TNF-α) and interleukins (ILs) in AA [3], [4], [5]. Meanwhile, decreased early hematopoietic growth factors (HGFs) produced by stromal cells diminish the process of hematopoiesis. There is increasing evidence suggesting that AA might be a syndrome characterized by stem/progenitor-cell disorders including HSCs and bone marrow mesenchymal stem cells (BM-MSCs).

Previous studies have demonstrated that HSCs from AA patients are damaged and defective in multiple biological properties and functions. Using clonogenic cultures *in vitro*, AA manifests as a bone marrow failure syndrome with marked reduction of various subpopulations of HSCs and common myeloid progenitors [6], [7], [8], [9]. Induced apoptosis of HSCs has been reported as a possible mechanism for the bone marrow failure in AA [10], [11], [12], [13]. Consistent with the above, gene expression profile of CD34^+^ cells from AA patients identified a large number of different genes expression, which belong mainly to: immune response, apoptosis, cell cycle and proliferation [14].

A microarray approach was also used to elucidate the complicated immune mechanisms of AA. Zeng et al [15] showed that there were 178 and 183 differentially expressed genes in CD4^+^ T cells and CD8^+^ T cells respectively from AA patients compared to healthy controls. A variety of aberrant cytokines and chemokines, including IFN-γ and –β, MIP2A and 3a, CCR2, CCL2, IL-8, IL-1B and IL-1RA, are likely to play important roles in the recruitment and activation of lymphocytes into cytotoxic effectors for marrow hematopoietic target cells in AA. The gene expression profiles further confirmed that the T cell is the major factor mediating the pathogenesis of AA. Both innate and adaptive immune responses of CD4^+^ and CD8^+^ T cells contribute to the immune-mediated HSCs destruction in AA. Meanwhile, it is important to elucidate whether aberrant immune damage BM-MSCs as well as HSCs are implicated in the development of AA.

As the key precursor cells of marrow microenvironment, BM-MSCs are of great importance in maintaining long-term hematopoiesis. MSCs differentiate into a variety of stromal cells which constitute the HSC niche. MSCs and differentiated stromal cells support hematopoiesis and regulate overall immune cells function to maintain hematopoietic and immune homeostasis. MSCs possess remarkable immune- suppressive properties of Th1 and CTLs [16], [17], [18]. Sporadic research has demonstrated that BM-MSCs from AA patients had poor proliferation and deficient immune suppression of mixed lymphocyte reaction (MLR) and IFN-γ release [19], [20]. However, a review of the limited investigations about MSCs could not fully explain the abnormal role of BM-MSCs as HSCs in AA.

Therefore, we performed this research to carry out a comprehensive analysis of BM-MSCs from AA patients. We tried to obtain more evidence for the failure of the marrow microenvironment in the pathogenesis of AA. We showed that BM-MSCs from AA patients were abnormal in: morphology, proliferation and clonogenic potential, multiple differentiations and apoptosis. The gene expression profile of BM-MSCs further confirmed the above abnormal biological properties and supplied significant evidence for the possible mechanisms of the destruction of the bone marrow microenvironment in AA.

## Materials and Methods

### Patients and controls

We analyzed bone marrow samples from 21 AA patients (12 men and 9 women) and 20 healthy controls (11 men and 9 women). The median age was 28.5 (range 15–57) years for AA patients and 32 (range 19–56) years for healthy controls. The diagnosis of AA was established by morphological examination of bone marrow and blood after exclusion of any other marrow failure syndromes, such as paroxysmal nocturnal hemoglobinuria, myelodysplastic syndrome, congenital bone marrow failure syndromes [21]. Additionally, secondary aplastic anemia following toxic exposure, infectious disease, pregnancy, autoimmune disorders, drugs and tumorous diseases according to the international criteria were also excluded. The degree of cellularity ranged from 18–25 percent in the diagnostic bone marrow specimens. All patients were newly diagnosed and were free from any antecedent, concurrent illness and infectious diseases. They had not been treated with any specific therapy including cyclosporine A and antithymocyte globulin (ATG) before enrollment. Healthy controls were allogeneic bone marrow donors identified by morphology examinations of bone marrow and blood.

This study was approved by the Institutional Review Board of the Chinese Academy of Medical Sciences and Peking Union Medical College. Bone marrow, peripheral blood and cord blood were obtained from donors after informed consent.

### Isolation and identification of BM-MSCs

Bone marrow mononuclear cells (BMMNCs) were isolated and cultured in Dulbecco's Modified Eagle Medium: Nutrient Mixture F-12 (D-MEM/F-12) (Gibco, Carlsbad, CA, USA) supplemented with 40% MCDB-201 (Sigma, St. Louis, USA), 2% fetal bovine serum (FBS) (Hyclone, Logan, UT, USA), 1×insulin-transferrin -selenium (ITS) (Gibco, Carlsbad, CA, USA), 10^−8^ M dexamethasone (Sigma, St. Louis, USA), 100 U/mL penicillin/streptomycin, 2 mM L-glutamine (Sigma, St. Louis, USA), 2 ng/mL human basic fibroblast growth factor (bFGF) and 10 ng/mL human epidermal growth factor (EGF) (PeproTech, Rocky Hill, NJ, USA). After 3 days, the culture medium was completely replaced and non-adherent cells were removed. At about 80–85% confluency, the adherent cells were detached by treatment with 0.125% trypsin and 0.1% ethylene diamine tetraacetic acid (EDTA) (Sigma, St. Louis, USA) and replanted at a 1∶2 dilution under the same culture conditions. At passage 3, adherent cells were identified by surface markers with monoclonal antibodies CD29, CD166, CD44, CD73, CD49e, CD34, CD90, CD45, HLA-DR, HLA-ABC (BD Pharmingen, San Jose, CA, USA) and CD105 (AbD SeroTec, Oxford, UK) using a FACScan flow cytometer (BD Biosciences, Mountain View, CA, USA).

### Morphology of BM-MSCs

BM-MSCs (P3) were cultured in D-MEM/F-12 medium and observed under an inverted microscope (OLYMPUS IX71S8F-2, Tokyo, Japan). Meanwhile, BM-MSCs (P3) were stained with β-tubulin for the morphology examination. BM-MSCs were first washed and fixed with 3.7% paraformaldehyde for 15 minutes and 100% cold ethanol for 10 minutes. Then, BM-MSCs were labeled with 1∶200 β-tubulin for 12 hours and 4, 6-diamino-2-phenylindole (DAPI) (Fluka, St. Louis, MO, USA) for 5 minutes. Finally, BM-MSCs were measured using a fluorescence confocal microscope (Leica TCS SP2, Leica Microsystems,Wetzlar, Germany).

### Differentiation capacity of BM-MSCs

BM-MSCs (P4) were induced to differentiate into osteoblasts and adipocytes by using the following procedure. The induction medium for osteogenesis was Iscove's Modified Dulbecco's Medium (IMDM) (Gibco, Carlsbad, CA, USA) supplemented with 10% FBS, 10^−7^ M dexamethasone, 0.2 mM ascorbic acid 2-phosphate, and 10 mM glycerol 2-phosphate (Sigma, St. Louis, USA). The induction medium for adipogenesis was IMDM supplemented with 10% FBS, 10^−6^ M dexamethasone, 0.5 mM 3-isobutyl-1- methylxanthine, 10 mg/mL insulin and 60 µM indomethacin (Sigma, St. Louis, USA). After 3 days, the culture medium was completely replaced. The medium was then changed twice weekly thereafter. After the predetermined culture time had elapsed, adipocytes were stained with Oil Red O, and the osteoblasts with von Kossa, alkaline phosphatase and alizarin red S assays (Sigma, St. Louis, USA).

### CFU-F assay and proliferation assay of BM-MSCs

The colony-forming unit-fibroblast (CFU-F) assay was performed after the first passage. Cells were seeded in D-MEM/F-12 medium supplemented with 20% FBS in six-well plates at a density of 1×10^2^ cells/well. The medium was replaced every three days. After incubation for 14 days, the flasks were washed twice, fixed with 100% methanol and stained with 3% crystal violet. Cell clusters consisting of at least 50 fibroblasts were scored as a CFU-F colony.

Cell proliferation was measured by incorporation of BrdU using cell proliferation enzyme-linked-immunosorbent assay (ELISA) (Roche Molecular Biochemicals, Mannheim, Germany) according to the manufacturer's instructions. BM-MSCs (P3) were seeded in triplicate in 96-well plates at a density of 2×10^3^ cells/well. The optical density (OD) values were determined in triplicate at 450 nm.

### Apoptosis of BM-MSCs

Apoptotic rate of BM-MSCs (P4) was evaluated using FITC Annexin V Apoptosis Detection Kit (BD Pharmingen, San Jose, CA, USA). BM-MSCs (1×10^6^ cells) were harvested, washed and incubated in the Annexin V-binding buffer. Annexin V-FITC and PI were added, followed by measurement using flow cytometry. The apoptotic cells included cells in early apoptosis (FITC Annexin V positive and PI negative) and in late apoptosis (both FITC Annexin V and PI positive).

### Affymetric Genechip assay

Total RNA was extracted from BM-MSCs(P3)using TRIzol reagent according to the manufacturer's instructions. To provide sufficient total RNA for processing, samples were pooled as outlined in a previous study by Zeng et al [14]. The detailed clinical features of AA patients are shown in Table 1. A RNA pool from 5 AA patients (equal amounts of RNA from each individual) was named pool-AA1, and pool-AA2 was obtained from another cohort of 3 AA patients. For controls, pool-N1 was prepared from 5 healthy individuals and pool-N2 from an additional 3 healthy individuals. In addition, pool-AA3 and pool-N3 were prepared from a further 3 AA patients and healthy controls respectively for real-time polymerase chain reaction (PCR) assay. Biotinylated cRNA synthesis, hybridization to human genome U133 plus 2.0 GeneChip microarrays, washing, staining, and scanning were performed as described in the standard Affymetrix protocol for Human Genome Arrays in CapitalBio Incorporation (Beijing, China). Quantitative real-time PCR was performed to confirm gene expression levels of RNA transcripts with sequence-specific oligonucleotide primers as described previously.

**Table 1:** Clinical features of patients with aplastic anemia.

Patient	Age/Sex	Diagnosis	Neutrophil (10^9^/L)	PLT (10^9^/L)	Ret (10^9^/L)	Groups
1	23/M	SAA	0.7	13	7.4	pool-AA1
2	19/M	SAA	0.2	6	12	pool-AA1
3	36/F	SAA	0.59	16	4.3	pool-AA1
4	15/M	SAA	0.59	15	15.9	pool-AA1
5	47/F	SAA	0.16	3	7.8	pool-AA1
6	19/M	SAA	0.7	5	17.1	pool-AA2
7	41/F	SAA	0.47	15	22.3	pool-AA2
8	23/M	SAA	0.15	21	8.5	pool-AA2
9	23/F	SAA	0.44	10	23.9	pool-AA3
10	44/M	SAA	0.4	19	33.4	pool-AA3
11	30/M	SAA	0.44	3	4.2	pool-AA3

The degree of cellularity ranged from 18–25 percent in the diagnostic bone marrow specimens. M indicated male. F indicated female.

### Quantitative real-time PCR

cDNA was prepared using oligo (dT) primer and Moloney murine leukemia virus reverse transcriptase (Promega, Madison, WI, USA). Primers (IL-6, leptin, CEBPD, GADD45B, CCNB1, TELO2 and PDGFD) were synthesized by Sangon Biotech Co. Ltd (Shanghai, China) (not shown). Quantitative real-time PCR was performed with 2×SYBR Green PCR Master Mix (Applied Biosystems, Foster, CA, USA) in the Applied Biosystems Gene Amp 7500 Sequence Detection System as described previously [22]. The cycle conditions were as follows: 95°C for 10 minutes and 40 cycles of 94°C for 15 seconds, 60°C for 1 minute. Relative quantities of target genes were determined for unknown samples by the comparative threshold cycle (delta CT) method and normalized to GAPDH quantities. All samples were run in triplicate.

### Statistics

Data was analyzed with SPSS 15.0 software. Results are presented as mean ± SD. The statistical differences between groups were evaluated by One-Way ANOVA and the differences in groups were evaluated by Paired-Samples T Test, defined as a value of *P*<0.05. For GeneChip analysis, mRNA expression level for BM-MSCs was determined in two steps as previously described [14]. Genes differentially expressed in BM-MSCs from AA were identified with at least a 2.0-fold change with respect to normal pools.

## Results

### Isolation and phenotype identification of BM-MSCs

BM-MSCs were isolated from all healthy controls and 17 of 21 AA patients for further investigations. BM-MSCs were harvested at passage 3 to analyze their immunophenotype using flow cytometry. BM-MSCs from both AA patients and healthy controls expressed: CD105 (SH2), CD73 (SH3), CD90, CD29, CD44, CD49e, CD166 and HLA-ABC, but lack expression of CD34, CD45 and HLA-DR (Table 2).

**Table 2:** Phenotype characteristics of BM-MSCs (n = 11).

Surface markers	Control (%)	AA (%)
CD34	-	-
CD45	-	-
CD73	99.71±0.16	99.55±0.03
CD105	87.45±5.19	75.88±22.28
CD90	83.77±1.85	89.55±1.95
CD44	99.63±0.06	99.61±0.11
CD29	99.52±0.17	99.64±0.07
CD49e	98.18±0.66	98.68±0.7
CD166	94.09±3.99	85.72±8.11
HLA-ABC	99.71±0.16	99.71±0.16
HLA-DR	-	-

### Morphology and multi-potential differentiation of BM-MSCs

**Figure 1:**
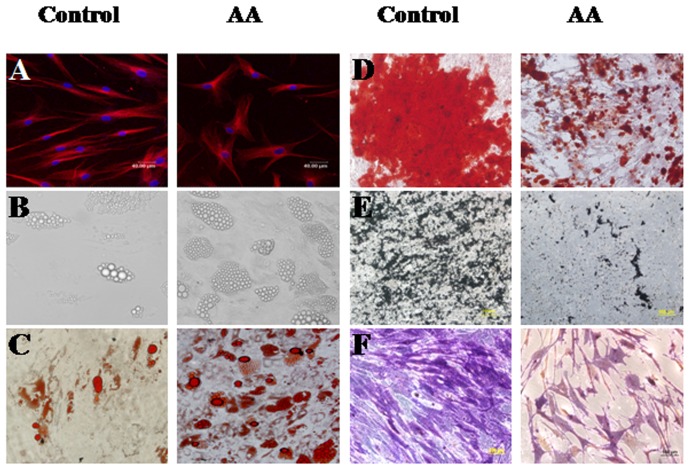
The representative morphology and multiple differentiation capacity of BM-MSCs from AA patients and healthy controls. The morphology of BM-MSCs was shown after staining with β-tubulin (**A**). The adipogenic differentiation capacity of BM-MSCs detected by un-staining (**B**) and positive staining of Oil Red O (**C**). The osteogenic differentiation capacity of BM-MSCs detected by positive staining of Allizarin Red (**D**), von Kossa (**E**) and ALP (**F**).

Both BM-MSCs formed a monolayer of bipolar spindle-like cells with a whirlpool-like array. However, BM-MSCs from AA patients seemed aberrant with irregular and ragged appearance after staining with β-tubulin under a fluorescence confocal microscope (Figure 1A). After induction with different conditional media, BM-MSCs could differentiate into osteoblasts and adipocytes as detected by positive staining of Allizarin Red, von Kossa, ALP and Oil Red O, respectively. Compared to healthy controls, BM-MSCs from AA were easily induced to differentiate into adipocyte lineage (Figure 1B and 1C), but it was more difficult to induce them to differentiate into osteoblasts (Figure 1D–F).

### Clonogenic and proliferation capacity of BM-MSCs

**Figure 2:**
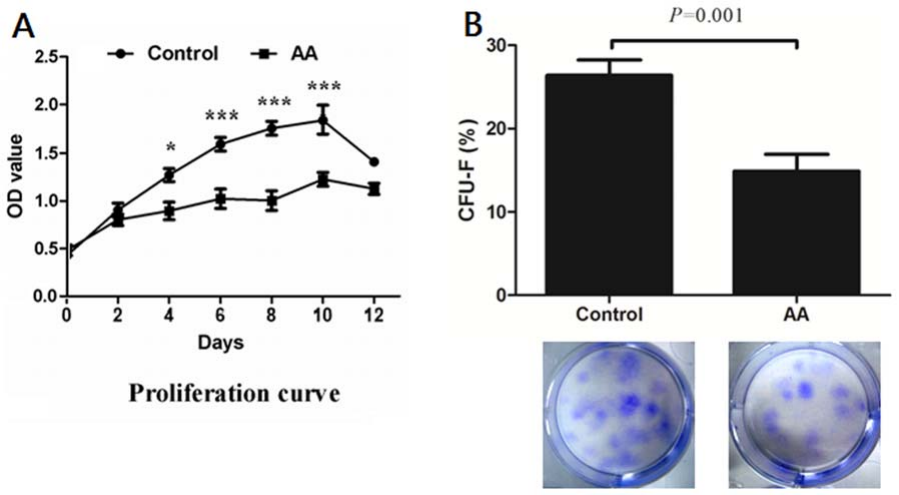
The proliferation and CFU-F capacity of BM-MSCs. (**A**) The proliferation rate of BM-MSCs were detected using BrdU-ELISA method after different incubation times. *P<0.05, ***P<0.001. (**B**) The clonogenic capacity was assessed by colony-forming unit-fibroblast (CFU-F) assay. Data represented mean ± SD (n = 10).

Cell proliferation was measured by incorporation of BrdU using ELISA. The OD values were detected after culture for 0, 2, 4, 6, 8, 10 and 12 days, respectively. As shown in Figure 2A, the proliferation rate of BM-MSCs from AA patients was significantly lower than that of healthy controls (4 days: *P*<0.05, 6 days: *P*<0.001, 8 days: *P*<0.001, 10 days: *P*<0.001). The clonogenic potential of BM-MSCs was assessed by CFU-F assay. In comparison with healthy controls, BM-MSCs from AA patients displayed lower clonogenic capacity *in vitro* (26.40±5.80% *vs* 14.90±6.40%, *P* = 0.001) (Figure 2B).

### Apoptosis rate of BM-MSCs

**Figure 3:**
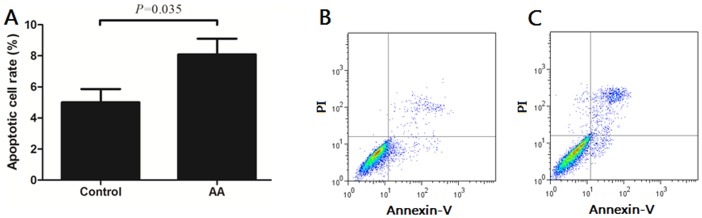
The apoptosis rate of BM-MSCs from AA patients and healthy controls. The apoptotic rate of BM-MSCs was assessed with Annexin V Apoptosis Detection Kit (**A**). The representative dotplots of apoptotic rate of BM-MSCs from healthy controls (**B**) and AA patients (**C**). Data represented mean ± SD (n = 10).

We further assessed the apoptotic cell rate with Annexin V Apoptosis Detection Kit. As shown in Figure 3A, the apoptotic cell rate of BM-MSCs from AA patients (8.09±3.22%) was higher than that of healthy controls (5.00±2.59%) (*P* = 0.035). The representative dotplots of apoptotic rate of BM-MSCs also showed that the number of apoptotic BM-MSCs from AA patients (Figure 3B) was higher than that of healthy controls (Figure 3C).

### Global view of gene expression profile of BM-MSCs

**Figure 4:**
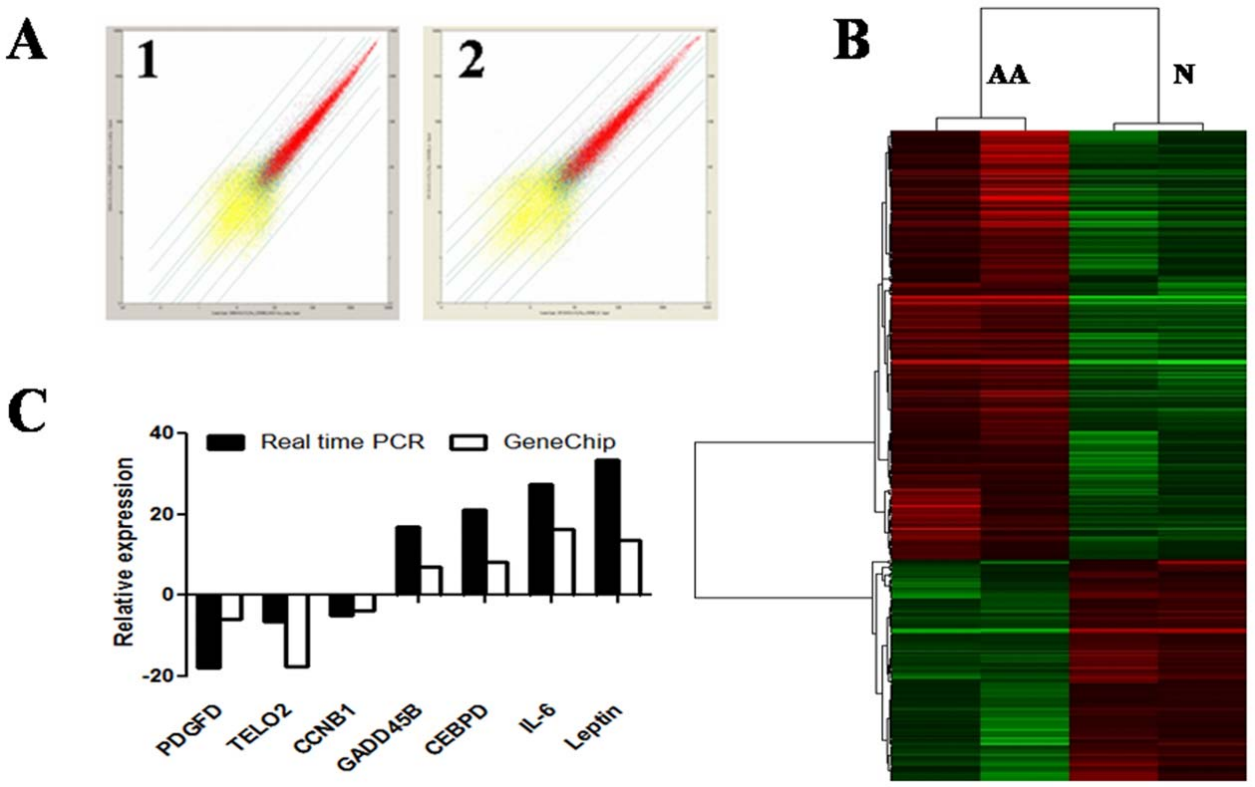
Global view of gene expression profile of BM-MSCs. Gene expression profile of BM-MSCs was determined using Affymetrix oligoarrays. (**A**) The scatter plot for two biological replicates. (**B**) The comparison of cluster data between AA patients and healthy controls. (**C**) Validation of GeneChip results by quantitative real-time PCR. Data were relative to the amount of GAPDH.

We analyzed the gene expression profile of BM-MSCs using Affymetrix oligoarrays (human genome U133 plus 2.0 GeneChip microarrays). To account for differences between individuals and to obtain adequate quantities of RNA for the analysis, we pooled equal amounts of BM-MSCs RNA from patients (pool-AA1 or pool-AA2) or healthy controls (pool-N1 or pool-N2) as previously described [14]. Genes differentially expressed in BM-MSCs from AA patients were identified with at least a 2.0-fold change with respect to healthy control pools. Meanwhile, quantitative real-time PCR (pool-N3 and pool-AA3) was performed in order to confirm the results of GeneChip profile. The selected genes expression in GeneChip detection were consistent with the results assessed using quantitative real-time PCR (Figure 4).

Compared to healthy controls, a total of 314 genes were differentially expressed in BM-MSCs from AA patients. Overall, 207 genes were up-regulated and 107 genes were down-regulated, which belonged to different functional categories and signal pathways. Up-regulated genes located in the range of 10.0–42.0 fold change (1.93%), 5.0–10.0 fold change (14.01%) and 2.0–5.0 fold change (84.06%). Down-regulated genes located in the range of 0–0.1 fold change (0.94%), 0.1–0.2 fold change (2.80%) and 0.2–0.5 fold change (96.26%). The top differential genes expression of BM-MSCs between AA patients and healthy controls are shown in Table 3 and 4.

**Table 3:** The top 10 differential up-regulated genes expression.

Gene	Gene title	Location	Transcript ID	Fold change
EIF1AY	eukaryotic translation initiation factor 1A, Y-linked	chrYq11.222	NM_004681	41.05
DDX3Y	DEAD box polypeptide 3, Y-linked	chrYq11	NM_001122665	36.08
USP9Y	ubiquitin specific peptidase 9, Y-linked	chrYq11.2	NM_004654	27.67
RPS4Y1	ribosomal protein S4, Y-linked 1	chrYp11.3	NM_001008	27.27
PTGS2	prostaglandin-endoperoxide synthase 2	chr1q25.2–q25.3	NM_000963	9.94
ID4	inhibitor of DNA binding 4	chr6p22-p21	NM_001546	9.24
SERPINB2	serpin peptidase inhibitor, clade B, member 2	chr18q21.3	NM_001143818	8.92
NR4A1	nuclear receptor subfamily 4, group A, member 1	chr12q13	NM_002135	8.22
TTTY15	testis-specific transcript, Y-linked 15	chrYq11.1	NR_001545	7.76
F2R	coagulation factor II (thrombin) receptor	chr5q13	NM_001992	7.32

**Table 4:** The top 10 differential down-regulated genes expression.

Gene	Gene title	Location	Transcript ID	Fold change
SIM1	single-minded homolog 1	chr6q16.3–q21	NM_005068	0.05
EMX2	empty spiracles homeobox 2	chr10q26.1	NM_004098	0.1
FABP4	fatty acid binding protein 4, adipocyte	chr8q21	NM_001442	0.14
IL13RA2	interleukin 13 receptor, alpha 2	chrXq13.1–q28	NM_000640	0.19
HLA-DRB1	major histocompatibility complex, class II, DR beta 1	chr6p21.3	NM_002124	0.2
HMGCS1	3-hydroxy-3-methylglutaryl-Coenzyme A synthase 1	chr5p14-p13	NM_001098272	0.21
FABP3	fatty acid binding protein 3, muscle and heart	chr1p33-p32	NM_004102	0.21
PHACTR2	phosphatase and actin regulator 2	chr6q24.2	NM_001100164	0.21
C13orf15	chromosome 13 open reading frame 15	chr13q14.11	NM_014059	0.21
EIF4A2	eukaryotic translation initiation factor 4A, isoform 2	chr3q28	NM_001967	0.22

### Differential signal pathways between BM-MSCs from AA patients and healthy controls

Differential genes were classified into significant signal pathways by using Kyoto Encyclopedia of Genes and Genomes Pathway database (KEGG) analysis. The differential signal pathways are shown according to the Q value in Table 5. The total genes and representative genes in respective signal pathways are also shown. The differential signal pathways included biosynthesis of steroids, cell cycle, adipocytokine signaling pathway, cell adhesion molecules (CAMs), transforming growth factor-beta (TGF-β) signaling pathway, hematopoietic cell lineage and apoptosis, etc. For example, CDC2, CDKN2A, CCND2, CCNA2 and CCNB1 were differently expressed in cell cycle pathway. Abnormal CNTNAP2, ITGB8, JAM3, ITGA8 and ICAM1 contributed to the process of the cell adhesion pathway. ID3, SMAD3, ID4, ID2, ID1 and SMAD7 played important roles in the TGF-β signaling pathway.

**Table 5:** Differential pathways of BM-MSCs between AA and healthy controls.

Pathways	Total genes	Q value	Representative gene title
Biosynthesis of steroids	13	0.0	EBP, SQLE, LSS, FDFT1, HMGCR
Cell cycle	14	1.0E-6	CDC2, CDKN2A, CCND2, CCNA2, CCNB1
Adipocytokine signaling pathway	8	2.17E-4	SOCS3, CPT1A, PRKAB2, TRADD, LEP
Cell adhesion molecules (CAMs)	14	3.94E-4	CNTNAP2, ITGB8, JAM3, ITGA8, ICAM1
TGF-beta signaling pathway	10	5.4E-4	ID3, SMAD3, ID4, ID2, ID1, SMAD7
Hematopoietic cell lineage	9	6.96E-4	KITLG, CD9, IL6, MME, CD36
Wnt signaling pathway	12	7.09E-4	SMAD3, PRICKLE1, CCND2, SFRP1, CTNNB1
ABC transporters - General	5	8.31E-4	ABCA6, ABCA8, ABCA13, ABCD3, ABCA9
Cytokine-cytokine receptor interaction	15	9.85E-4	CXCL2, CXCL1, CXCL6, CXCL5, VEGFC
Antigen processing and presentation	9	0.003	HLA-DRB3, HLA-DQA, NFYB1, HLA-DRA
Tryptophan metabolism	7	0.003	ACAT2, MID1, DHCR24, MYLIP, MIB1
Leukocyte transendothelial migration	7	0.003	GNAI3, IL8, JAM3, ICAM1, CTNNA1
Fatty acid biosynthesis	2	0.004	THEDC1, FASN
Complement and coagulation cascades	6	0.005	SERPING1, C1S, C7, F2R, CD46
Vitamin B6 metabolism	2	0.007	MTMR1, PSAT1
Fatty acid metabolism	4	0.008	ACAT2, CPT1A, ADH1A, ADH1C
Tight junction	6	0.01	CSDA, GNAI3, JAM3, CTNNA1, MAGI1
Adherens junction	4	0.023	SMAD3, LMO7, CTNNA1, CTNNB1
Epithelial cell signaling in Hp infection	3	0.025	CXCL1, IL8, JAM3
ECM-receptor interaction	6	0.032	ITGB8, HMMR, ITGA8, CD36
Apoptosis	5	0.039	CYCS, TRADD, TNFSF10, ATM
Glycerolipid metabolism	3	0.039	ADH1A, ADH1C, LIPG
MAPK signaling pathway	9	0.04	STK4, DIT3, FGFR2, BDNF, NR4A1
Insulin signaling pathway	5	0.042	SOCS3, GYS1, PRKAB2, PRKY, FASN
Gap junction	6	0.048	CDC2, GNAI3, PRKY, PLCB1

### Differential biological processes between BM-MSCs from AA patients and healthy controls

Differential genes were also classified into a variety of categories by using GO mapping analysis. Here, we showed the biological process categories according to the Q value in Table 6. The total genes and representative genes in related biological process are also shown. The differential biological processes included cholesterol biosynthesis, fatty acid biosynthesis, negative regulation of transcription factor activity, negative regulation of cell proliferation, regulation of transcription, etc. For example, ID1, ID3 and ID2 were differently expressed in the process of negative regulation of transcription factor activity. Abnormal IL-6, MDM2, GPNMB, IL-8 and EREG affected the biological process of negative regulation of cell proliferation. CXCL5, CXCL2, IL8, CXCL6 and CXCL1 played important roles in the process of BM-MSCs chemotaxis.

**Table 6:** Differential biological process associated with BM-MSCs.

Go Term	Total genes	Q value	Representative gene title
Cholesterol biosynthesis	16	0.0	DHCR7, IDI1, FDPS, DHCR24
Fatty acid biosynthesis	10	0.0	PRKAB2,PTGS1, SCD, FASN
Negative regulation of transcription factor activity	5	7.9E-5	ID1, ID3, ID2
Negative regulation of cell proliferation	12	8.5E-5	IL6, MDM2, GPNMB, IL8, EREG
Regulation of transcription, DNA-dependent	62	1.17E-4	BHLHB2, MEOX2, MEOX2, ZNF655
Anti-apoptosis	8	6.23E-4	DDAH2, MALT1, HIPK3, IER3, BNIP2
Complement activation, classical pathway	5	9.76E-4	C1S, SERPING1, C7, CD46
Keratinocyte differentiation	4	0.002	PTGS1, EREG, PTGS2
Cell-cell signaling	19	0.002	NDP, NAMPT, IL6, LEP, AREG
Negative regulation of cell adhesion	3	0.002	CD164, Q8NFD8_HUMAN, ADAMDEC
Regulation of cyclin-dependent protein kinase activity	4	0.003	CCNA2, CCNE2, CDKN2A, RGC32
Cell division	4	0.004	CAPG, CDC2, CCNB1, CCND2, CCNA2
Cytokinesis	4	0.005	DIAPH2, ANLN, PRC1
Mitosis	11	0.006	PBK, CAPG, CDC2, CCNB1, CCNA2
Chemotaxis	7	0.008	CXCL5, CXCL2, IL8, CXCL6, CXCL1
Positive regulation of mitosis	3	0.008	EREG, NUSAP1
Positive regulation of I-kB kinase/NF-kB cascade	6	0.009	MALT1, CASP1, TNFSF10, TRADD
Regulation of progression through cell cycle	15	0.012	CDC2, CCNB1, MDM2, CCND2, FOSB
TGF-beta receptor signaling pathway	3	0.012	SMAD7, LTBP2, SMAD3
Positive regulation of cell proliferation	8	0.012	NAMPT, IL6, CXCL5, TSHR, TTK
Angiogenesis	5	0.015	IL8, EREG, ANG, ANGL4_HUMAN
Innate immune response	5	0.02	C1S, SERPING1, C7, CD46
Negative regulation of lipoprotein lipase activity	2	0.021	ANGL4_HUMAN
Cytokinesis after mitosis	2	0.021	NUSAP1
Cell cycle checkpoint	3	0.024	RAD1, CCNE2, CDKN2A
Cell cycle arrest	4	0.024	DHCR24, IL8, CDKN2A, Q8N9B5
Protein ubiquitination	3	0.032	MYLIP, MDM2, FBXO9
Negative regulation of transcription	3	0.032	NAB1, EREG, ID1, ID4, ID3

### Differential gene expression associated with the abnormal characteristics of BM-MSCs from AA patients

**Figure 5:**
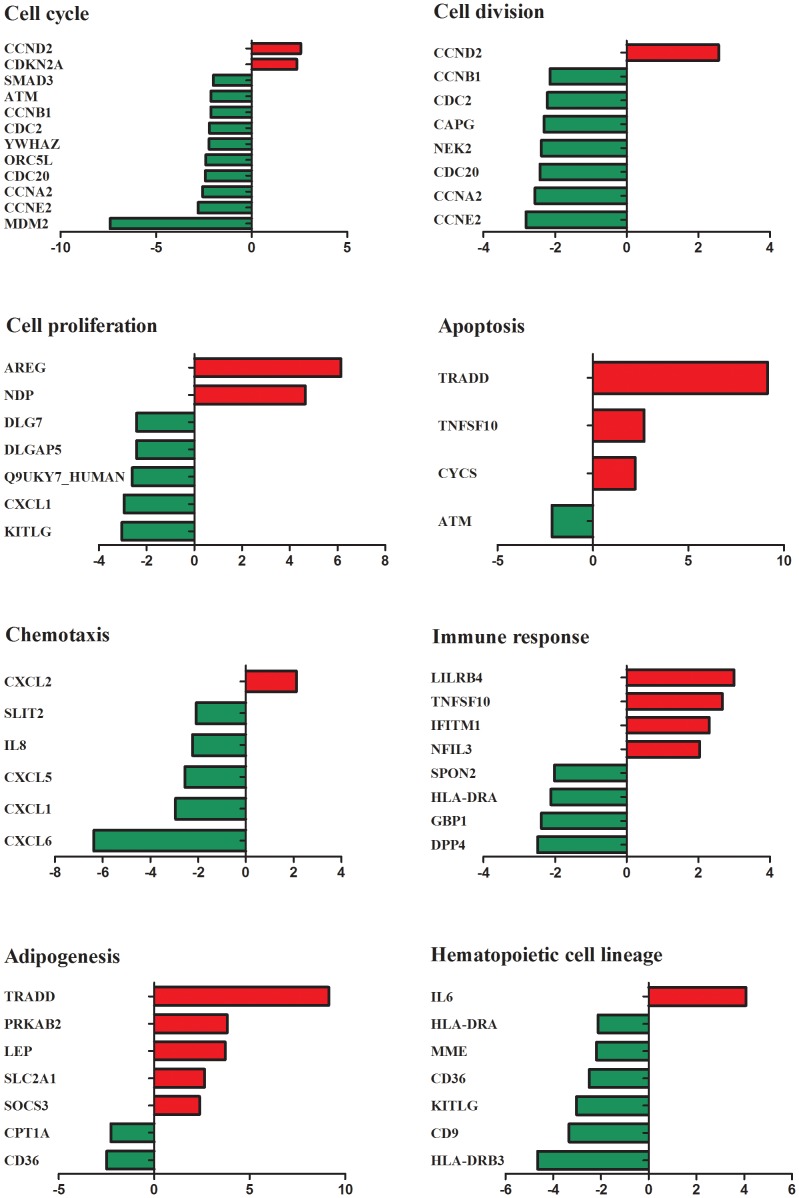
Gene expression patterns of BM-MSCs from AA patients in key biological signal pathways. Gene expression patterns were grouped and displayed in the following categories: cell cycle, cell division, cell proliferation, apoptosis, chemotaxis, immune response, adipogenesis, and hematopoietic cell lineage. Relative genes expression were analyzed and compared between AA patients and healthy controls. Genes differentially expressed in BM-MSCs from AA patients were identified with at least a 2.0-fold change with respect to healthy control pools. The up-regulated genes are shown in red while down-regulated genes are shown in green.

Our previous data confirmed that BM-MSCs from AA patients were aberrant in multiple biological features including morphology, proliferation and differentiation potential, and apoptosis. Therefore, we paid more attention to the related signal pathways such as cell cycle, cell division, cell proliferation, apoptosis, chemotaxis, immune response, adipognesis and hematopoietic cell lineage. Many genes were found to be dysregulated, which was consistent with our previous results (Figure 5). Most genes implicated in cell cycle, cell division, proliferation, chemotaxis and hematopoietic cell lineage were down-regulated. In cell cycle, CCND2 and CDKN2A were up-regulated while SMAD3, ATM, CCNB1, CDC2, YWHAZ, ORC5L, CDC20, CCNA2, CCNE2 and MDM2 were down-regulated in AA. In cell division, most genes (CCNB1, CDC2, CAPG, NEK2, CDC20, CCNA2 and CCNE2) were down-regulated except for CCND2. In cell proliferation, DLG7, DLGAP5, Q9UKY7_HUMAN, CXCL1 and KTTLG were decreased except for NDP and AREG. In chemotaxis, SLIT2, IL8, CXCL5, CXCL1 and CXCL6 were down-regulated while CXCL2 were up-regulated. In hematopoietic cell lineage, HLA-DRA, MME, CD36, KITLG, CD9 and HLA-DRB3 were down-regulated while IL6 was up-regulated. However, more genes implicated in apoptosis, immune response and adipogenesis were up-regulated in BM-MSCs from AA patients. In the process of apoptosis, TRADD, TNFSF10 and CYCS were up-regulated except for ATM. In immune response, LILRB4, RNFSF10, IFTTM1 and NFIL3 were increased while SPON2, HLA-DRA, GBP1 and DPP4 were decreased. In adipogenesis, most genes (TRADD, PRKAB2, LEP, SLC2A1 and SOCS3) were up-regulated except for CPT1A and CD36. In brief, aberrant immune response might destroy BM-MSCs resulting in disabled precursors of bone marrow microenvironment with increased apoptosis, defective biological functions and abnormal differentiation in the development of AA.

## Discussion

The present study aimed to elucidate the biological features and differential gene expression profile of bone marrow MSCs from AA patients. First, we investigated the differences in morphology, immunophenotype markers, proliferation, differentiation, CFU-F and apoptosis of BM-MSCs between AA patients and healthy controls. Secondly, we analyzed the differences in the gene expression profile of BM-MSCs. We demonstrated that BM-MSCs from AA patients were multiply defective in biological properties, which were consistent with the differential gene expression profile.

Both HSCs and MSCs are key stem cells responsible for normal hematopoiesis in the bone marrow. HSCs maintain hematopoiesis through self-renewal and differentiation. MSCs provide the physical structure of the microenvironment for HSCs and interact with HSCs by cell-cell contact and secreting HGFs to support hematopoiesis. Previous investigations have demonstrated that HSCs are defective both in biological features and functions with decreased counts, quality and gene expression profile in AA [1], [2]. In comparison with HSCs little is known about the pathological nature of BM-MSCs in AA, although several investigators have recently provided some evidence that the biological properties of BM-MSCs in AA were abnormal [19], [20]. Therefore, we performed this research to demonstrate whether MSCs, as the key precursor stem cells of marrow microenvironment, are damaged following the destruction of HSCs, and whether abnormal MSCs aggravate bone marrow failure in the development of AA. Consistent with previous studies [19], we observed that there was no difference in immunophenotype markers of BM-MSCs between AA patients and healthy controls. We found that BM-MSCs from AA patients were aberrant with irregular and ragged appearance. We also showed reduced proliferation and clonogenic potential of BM-MSCs from AA patients. Interestingly, BM-MSCs from AA patients were susceptible to be induced to differentiate into adipocytes but more difficult to differentiate into osteoblasts, which may cause increased adipogenesis and abnormal formulation of the HSCs niche. More severely, there was an increased apoptotic rate of BM-MSCs from AA patients, which surely led to the reduction of BM-MSCs accompanied with damaged HSCs. Thus, we considered that both BM-MSCs and HSCs were present with decreased counts, reduced proliferation and clonogenic potential and increased apoptosis during the development of AA.

To further explore the mechanisms that BM-MSCs from AA patients were defective in biological properties, we undertook GeneChip analysis of BM-MSCs. 314 genes implicated in cell cycle, cell proliferation, differentiation, apoptosis, hematopoiesis and immune response were markedly differentially expressed between BM-MSCs from AA patients and healthy controls. The abnormal gene expression profile further confirmed the aberrant biological features of BM-MSCs, which was consistent with those of CD34^+^ cells in AA patients [14]. Both BM-MSCs and CD34^+^ cells showed similar gene expression profile in the functional categories of cell cycle, proliferation, apoptosis, hematopoietic cell lineage and immune response. A number of genes involved in the signal pathways for cell cycle (SMAD3, ATM, CCNB1, CDC2, YWHAZ, ORC5L, CDC20, CCNA2, CCNE2 and MDM2), cell division (CCNB1, CDC2, CAPG, NEK2, CDC20, CCNA2 and CCNE2) and cell proliferation (DLG7, DLGAP5, Q9UKY7_HUMAN, CXCL1 and KTTLG) were increased in AA patients. These abnormal genes could reduce the signal transduction of MSCs self-duplication and differentiation into terminal stromal cells in the bone marrow. Down-regulated genes correlated with chemotaxis might lead to decreased movement to the appropriate place. Furthermore, apoptosis-related genes (TRADD, TNFSF10 and CYCS) could enhance apoptosis through death receptor signal pathways. Damaged MSCs could not conduct their effective immune-regulation potential, and contribute to defective HSCs to maintain long-term hematopoiesis. It is well known that aplastic anemia is also characterized by fatty bone marrow and filmy cortical bone besides hypoplasia and pancytopenia [23], [24]. This related phenomenon could be demonstrated through increased adipogenesis mediated by genes (TRADD, PRKAB2, LEP, SLC2A1 and SOCS3) and decreased osteogenesis. Enhanced adipogenesis and reduced osteogenesis destroyed the formation of the bone marrow niche.

The aberrant properties and gene expression profile of BM-MSCs from AA patients hinted that their functions were abnormal in AA. Normally, HSCs maintain long-term hematopoiesis through self-duplication, and differentiate into multiple lineages and mature to terminal blood cells. MSCs support the process of hematopoiesis of HSCs by cell-cell contact and secreting HGFs. MSCs possess remarkable immunosuppressive properties of T cells, NK cells and DCs [17], [18]. Thus, the disorder of genes associated with hematopoiesis and immune response attenuated the functions of BM-MSCs in AA. Our data in other studies further confirmed this hypothesis (data not shown). We found that BM-MSCs from AA patients had reduced potential to amplify CD34^+^ cells and support the process of megakaryocytopoiesis. BM-MSCs from AA patients were reduced in suppressing the proliferation potential of CD4^+^ cells, TNF-α and IFN-γ production by CD4^+^ cells, and promoting Tregs expansion [25]. When BM-MSCs were damaged and became defective, the bone marrow suffered more severe losses in maintaining immune homeostasis which further aggravated the aberrant immunity in AA. Gene expression profile uncovered that genes implicated in immune response and hematopoiesis were dysregulated. These results provided the evidence that co-injection of MSCs and HSCs would enhance the reconstitution of hematopoiesis in AA patients, which has been confirmed in recent clinical applications [26], [27].

In summary, the present study has demonstrated that differential gene expression profile was consistent with multiple defects in biological properties of BM-MSCs from AA patients. These defects might finally lead to aggravating the bone marrow failure. However, the pathogenesis of AA is complicated. Further studies are required to determine how the defects of BM-MSCs arise in the development of aplastic anemia, and to develop effective therapeutic strategies such as transplantation of MSCs alone or co-transplantation with HSCs.

## References

[1] Young NS, Calado RT, Scheinberg P (2006) Current concepts in the pathophysiology and treatment of aplastic anemia. Blood 108: 2509–2519. 16778145 10.1182/blood-2006-03-010777 PMC1895575

[2] Li JP, Zheng CL, Han ZC (2010) Abnormal immunity and stem/progenitor cells in acquired aplastic anemia. Crit Rev Oncol Hematol 75: 79–93. 20045349 10.1016/j.critrevonc.2009.12.001

[3] Zoumbos NC, Gascon P, Djeu JY, Young NS (1985) Interferon is a mediator of hematopoietic suppression in aplastic anemia in vitro and possibly in vivo. Proc Natl Acad Sci U S A 82: 188–192. 3918301 10.1073/pnas.82.1.188 PMC396997

[4] Verma A, Deb DK, Sassano A, Kambhampati S, Wickrema A, et al (2002) Cutting edge: activation of the p38 mitogen-activated protein kinase signaling pathway mediates cytokine-induced hemopoietic suppression in aplastic anemia. J Immunol 168: 5984–5988. 12055203 10.4049/jimmunol.168.12.5984

[5] Sloand E, Kim S, Maciejewski JP, Tisdale J, Follmann D, et al (2002) Intracellular interferon-gamma in circulating and marrow T cells detected by flow cytometry and the response to immunosuppressive therapy in patients with aplastic anemia. Blood 100: 1185–1191. 12149196 10.1182/blood-2002-01-0035

[6] Maciejewski JP, Selleri C, Sato T, Anderson S, Young NS (1996) A severe and consistent deficit in marrow and circulating primitive hematopoietic cells (long-term culture-initiating cells) in acquired aplastic anemia. Blood 88: 1983–1991. 8822917

[7] Manz CY, Nissen C, Wodnar-Filipowicz A (1996) Deficiency of CD34+ c-kit+ and CD34+38- hematopoietic precursors in aplastic anemia after immunosuppressive treatment. Am J Hematol 52: 264–274. 8701944 10.1002/(SICI)1096-8652(199608)52:4<264::AID-AJH5>3.0.CO;2-Q

[8] Schrezenmeier H, Jenal M, Herrmann F, Heimpel H, Raghavachar A (1996) Quantitative analysis of cobblestone area-forming cells in bone marrow of patients with aplastic anemia by limiting dilution assay. Blood 88: 4474–4480. 8977239

[9] Rizzo S, Scopes J, Elebute MO, Papadaki HA, Gordon-Smith EC, et al (2002) Stem cell defect in aplastic anemia: reduced long term culture-initiating cells (LTC-IC) in CD34+ cells isolated from aplastic anemia patient bone marrow. Hematol J 3: 230–236. 12391540 10.1038/sj.thj.6200187

[10] Philpott NJ, Scopes J, Marsh JC, Gordon-Smith EC, Gibson FM (1995) Increased apoptosis in aplastic anemia bone marrow progenitor cells: possible pathophysiologic significance. Exp Hematol 23: 1642–1648. 8542959

[11] Timeus F, Crescenzio N, Doria A, Foglia L, Linari A, et al (2005) Flow cytometric evaluation of circulating CD34+ cell counts and apoptotic rate in children with acquired aplastic anemia and myelodysplasia. Exp Hematol 33: 597–604. 15850838 10.1016/j.exphem.2005.02.005

[12] Callera F, Falcao RP (1997) Increased apoptotic cells in bone marrow biopsies from patients with aplastic anaemia. Br J Haematol 98: 18–20. 9233557 10.1046/j.1365-2141.1997.1532971.x

[13] Killick SB, Cox CV, Marsh JC, Gordon-Smith EC, Gibson FM (2000) Mechanisms of bone marrow progenitor cell apoptosis in aplastic anaemia and the effect of anti-thymocyte globulin: examination of the role of the Fas-Fas-L interaction. Br J Haematol 111: 1164–1169. 11167757 10.1046/j.1365-2141.2000.02485.x

[14] Zeng W, Chen G, Kajigaya S, Nunez O, Charrow A, et al (2004) Gene expression profiling in CD34 cells to identify differences between aplastic anemia patients and healthy volunteers. Blood 103: 325–332. 14504100 10.1182/blood-2003-02-0490

[15] Zeng W, Kajigaya S, Chen G, Risitano AM, Nunez O, et al (2004) Transcript profile of CD4+ and CD8+ T cells from the bone marrow of acquired aplastic anemia patients. Exp Hematol 32: 806–814. 15345281 10.1016/j.exphem.2004.06.004

[16] Uccelli A, Moretta L, Pistoia V (2008) Mesenchymal stem cells in health and disease. Nat Rev Immunol 8: 726–736. 19172693 10.1038/nri2395

[17] Chen X, Armstrong MA, Li G (2006) Mesenchymal stem cells in immunoregulation. Immunol Cell Biol 84: 413–421. 16869941 10.1111/j.1440-1711.2006.01458.x

[18] Rasmusson I (2006) Immune modulation by mesenchymal stem cells. Exp Cell Res 312: 2169–2179. 16631737 10.1016/j.yexcr.2006.03.019

[19] Bacigalupo A, Valle M, Podesta M, Pitto A, Zocchi E, et al (2005) T-cell suppression mediated by mesenchymal stem cells is deficient in patients with severe aplastic anemia. Exp Hematol 33: 819–827. 15963858 10.1016/j.exphem.2005.05.006

[20] Chao YH, Peng CT, Harn HJ, Chan CK, Wu KH (2010) Poor potential of proliferation and differentiation in bone marrow mesenchymal stem cells derived from children with severe aplastic anemia. Ann Hematol 89: 715–723. 20084382 10.1007/s00277-009-0892-6

[21] Marsh JC, Ball SE, Cavenagh J, Darbyshire P, Dokal I, et al (2009) Guidelines for the diagnosis and management of aplastic anaemia. Br J Haematol 147: 43–70. 19673883 10.1111/j.1365-2141.2009.07842.x

[22] Li J, Zhao Q, Xing W, Feng J, Wu H, et al (2011) Interleukin-27 enhances the production of tumour necrosis factor-alpha and interferon-gamma by bone marrow T lymphocytes in aplastic anaemia. Br J Haematol 153: 764–772. 21506940 10.1111/j.1365-2141.2010.08431.x

[23] Steiner RM, Mitchell DG, Rao VM, Murphy S, Rifkin MD, et al (1990) Magnetic resonance imaging of bone marrow: diagnostic value in diffuse hematologic disorders. Magn Reson Q 6: 17–34. 2200500

[24] Negendank W, Weissman D, Bey TM, de Planque MM, Karanes C, et al (1991) Evidence for clonal disease by magnetic resonance imaging in patients with hypoplastic marrow disorders. Blood 78: 2872–2879. 1954375

[25] Li JP, Lu SH, Yang SG, Xing W, Feng JM, et al (2012) Impaired immunomodulatory ability of bone marrow mesenchymal stem cells on CD4+ T cells in aplastic anemia. Results in Immunology 2: 142–147. 24371578 10.1016/j.rinim.2012.07.002 PMC3862346

[26] Jaganathan BG, Tisato V, Vulliamy T, Dokal I, Marsh J, et al (2010) Effects of MSC co-injection on the reconstitution of aplastic anemia patient following hematopoietic stem cell transplantation. Leukemia 24: 1791–1795. 20724985 10.1038/leu.2010.164

[27] Wang H, Wang Z, Xue M, Liu J, Yan H, et al (2010) Co-transfusion of haplo-identical hematopoietic and mesenchymal stromal cells to treat a patient with severe aplastic. Cytotherapy 12: 563–565. 20380540 10.3109/14653241003695059

